# HTR for Greek Historical Handwritten Documents

**DOI:** 10.3390/jimaging7120260

**Published:** 2021-12-02

**Authors:** Lazaros Tsochatzidis, Symeon Symeonidis, Alexandros Papazoglou, Ioannis Pratikakis

**Affiliations:** Visual Computing Group, Department of Electrical and Computer Engineering, Democritus University of Thrace, 67100 Xanthi, Greece; ltsochat@ee.duth.gr (L.T.); ssymeoni@ee.duth.gr (S.S.); alpapazoglou@hotmail.com (A.P.)

**Keywords:** handwritten text recognition, convolutional neural networks, recurrent neural networks, gated recurrent unit, document image dataset

## Abstract

Offline handwritten text recognition (HTR) for historical documents aims for effective transcription by addressing challenges that originate from the low quality of manuscripts under study as well as from several particularities which are related to the historical period of writing. In this paper, the challenge in HTR is related to a focused goal of the transcription of Greek historical manuscripts that contain several particularities. To this end, in this paper, a convolutional recurrent neural network architecture is proposed that comprises octave convolution and recurrent units which use effective gated mechanisms. The proposed architecture has been evaluated on three newly created collections from Greek historical handwritten documents that will be made publicly available for research purposes as well as on standard datasets like IAM and RIMES. For evaluation we perform a concise study which shows that compared to state of the art architectures, the proposed one deals effectively with the challenging Greek historical manuscripts.

## 1. Introduction

Offline handwritten text recognition (HTR) in historical documents has become an attractive research field in computer vision, as it enables us to access our written past. The motivation for this work is the analysis of historical texts from the Greek Byzantine literature tradition, spanning between the fourth and the fifteenth century. The language in these texts is not homogeneous throughout the entire period, although an influence of the classical Greek language is prominent. Additionally, it corresponds neither to the spoken language of that time nor to the modern version of Greek used nowadays. However, the study of these sources is important as it provides access to even older texts that were retained throughout the centuries, being copied by the scribes of the Byzantine empire.

Several challenges are present for HTR systems targeting the specified era, caused by the age of the historical manuscripts that affects the clarity of the writing and the image quality in general. The language used in the writing results in increased complexity due to the multitude of diacritics, punctuation and abbreviating symbols that were used, leading to an increased character set compared to modern languages. The complexity is further increased by the fact that the content of such documents is unconstrained and might have been created by multiple writers.

In this paper, we present an OctCNN-BGRU (Octave Convolutional Neural Network-Bidirectional Gated Recurrent Units) architecture inspired by [1,2,3], with a focused goal of the transcription of Greek historical manuscripts. The contribution of this work is two-fold: first, a new architecture is proposed that employs Octave convolutions in the encoding stage to achieve a combination of higher- and lower-scale features in each layer; second, three newly created collections are presented, providing the means for the evaluation of our methodology and future research as well. Furthermore, to enable comparison with state of the art, we extended experimentation to three already available datasets, namely EPARCHOS [4], IAM [5] and RIMES [6].

The remainder of this paper is structured as follows: a thorough analysis of the state of the art is presented in Section 2. In Section 3, the proposed methodology is described in detail. Section 4 presents the experimental work and Section 5 concludes this research and outlines future directions.

## 2. Related Work

In offline HTR, recognition is based upon an input image of text, corresponding to either a text line or a document. In the latter case, a segmentation stage is also required to isolate text lines of the document and process them independently. There is a large number of studies that tackle this problem using deep learning methods, where recurrent neural networks have become a key component, such as long short-term memory (LSTM) [7] and gated recurrent unit (GRU) [8] networks.

An early approach in this direction is the work of Voigtlaender et al. [9] who developed an efficient GPU implementation of multidimensional LSTM [10] network and their research was focused on the depth and width of the architecture. Additionally, in their implementation training time is greatly reduced by processing the input in a diagonal-wise fashion.

The work of Puigcerver [2] introduced the Convolutional Recurrent Neural Network (CRNN) approach that replaced the two-dimensional recurrent blocks with a feature extraction CNN stage and a one-dimensional LSTM stage that processes each column of the image sequentially. This results in a reduced memory footprint while it increases the amount of calculation that can be parallelized, resulting in an efficient network architecture. Additionally, dropout and batch normalization were used in both stages, which increased performance, in accordance with the findings of Phamet al. [11].

Bluche and Messina [12] followed the aforementioned paradigm, with the main contribution being the use of convolutional gates [13] in the encoder part, which enables hierarchical context-sensitive feature extraction. For the decoding part, bidirectional one-dimensional LSTMs have been employed, that are being adapted to the different languages considered for testing. Similarly, de Sousa Neto et al. [14] used a combination of a convolutional encoder, based on Gated-CNN architecture, and a decoder with the addition of Bidirectional Gated Recurrent Units (BGRU) in the place of LSTMs. They also increased the number of layers in the encoder and incorporated dropout and the PReLU activation function. Using a similar network, Retsinas et al. [15] applied a semi-supervised approach to adapt an already trained network to the style of a specific test set, by formulating a loss functions that applies a weighting on each sample.

Motivated by transformers’ success in the Natural Language Processing (NLP) domain, specific efforts have been reported using Visual Transformers. In particular, Kang et al. [16] presented a new architecture utilizing multihead self-attention layers to handle image character recognition and decoding of language character sequences. In the same manner, Wick et al. [17] proposed a two-stage approach using both a CNN and a transformer-based encoder/decoder along with a voter to combine and extract the two predicted sequences. Finally, Wick et al. [18] proposed a combination network for HTR with a CNN/LSTM-encoder and a transformer-decoder with inserted mutual attention layers as a language model.

## 3. Methodology

**Figure 1:**
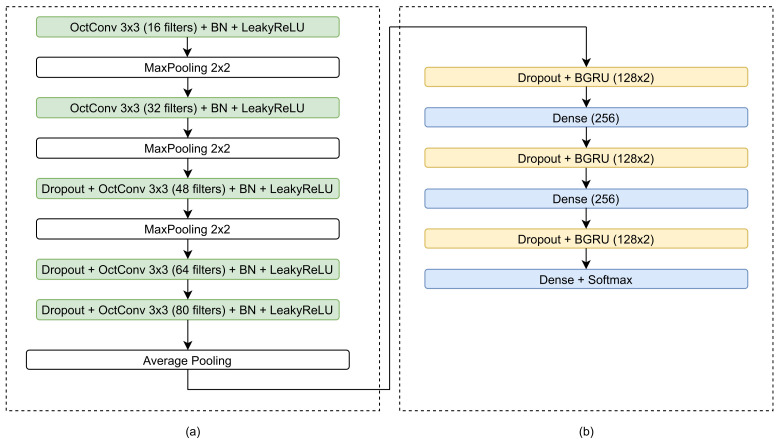
Schematic diagram of the proposed architecture, consisting of (**a**) the CNN stage and (**b**) the recurrent stage.

The overall proposed architecture consists of an image preprocessing module that feeds an OctCNN-BGRU. The proposed architecture, as shown in Figure 1, consists of a CNN stage for feature extraction and a recurrent stage for feature decoding into a probability vector corresponding to the different character classes. Each text line of the document is presegmented and processed separately, in a bidirectional manner. Each of the stages is presented in detail in the following sections.

### 3.1. Preprocessing

The preprocessing of the input images utilized in the proposed pipeline aims to standardize images from different sources and writers. Towards this end, Illumination Compensation [19] was used to remove shadows and balance brightness/contrast along a text line image. Firstly, the image undergoes a contrast enhancement followed by an edge detection method leading to the detection of the text area. Next, the background image is isolated by subtracting the detected text, and is used to assess the light distribution of the document. Finally, the initial image is balanced by adjusting each pixel value according to the light distribution.

As a next step, deslanting is applied, based on the work of Vinciarelli and Luettin [20], to soften the cursive style that may occur during handwriting, affecting the slope of the line and the slant of the letters. For the slope removal, the core region of a line is isolated by calculating a threshold on the horizontal density distribution histogram. Then, the image is rotated in order to eliminate the angle of the baseline with the *x*-axis. Slant correction is based on the hypothesis that the word is deslanted when the number of columns containing a continuous stroke is maximized. Towards this end, multiple shear transformations are applied to the image. For each vertical line of the transformed image, a histogram of the number of pixels belonging to text divided by their maximum distance is calculated. Finally, the version of the image with the maximum histogram energy is retained.

**Figure 2:**
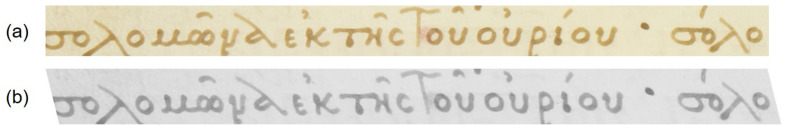
An example text line image (**a**) before and (**b**) after the preprocessing stage.

In Figure 2, the initial and the preprocessed versions of an example text line image are shown.

### 3.2. Octave-CNN Architecture

The octave-convolution operation (OctConv), introduced in [3], is a drop-in replacement for the convolution operation in a CNN architecture, which involves processing the input in two different scales, aiming to capture both high- and low-frequency patterns. Towards this end, the input feature map *X* is factorized into two portions along the channel axis, so that $X=\{X^{H},X^{L}\}$, resulting in two feature maps that capture fine- and low-detailed information. Subsequently, a new convolution operator is used to operate on this representation resulting in the layer output $Y=\{Y^{H},Y^{L}\}$, as defined in the following equations:

(1) $$Y^{H}=f\left(X^{H},W^{H\overset{}{\to }H}\right)+\mathrm{upsample}\left(f\left(X^{L},W^{L\overset{}{\to }H}\right),2\right)$$

(2) $$Y^{L}=f\left(X^{L},W^{L\overset{}{\to }L}\right)+f\left(\mathrm{pool}\left(X^{H},2\right),W^{H\overset{}{\to }L}\right)$$

where f(X,W) denotes convolution of *X* with the kernel *W* followed by an activation function, pool(X,k) denotes average pooling with kernel size *k* and upsample(X,k) denotes upsampling by a factor *k*. The partitioning of channels into high- and low-frequency features, that takes place in each OctConv layer, is configured by a hyperparameter α, that affects the number of convolution kernels for each band.

**Figure 3:**
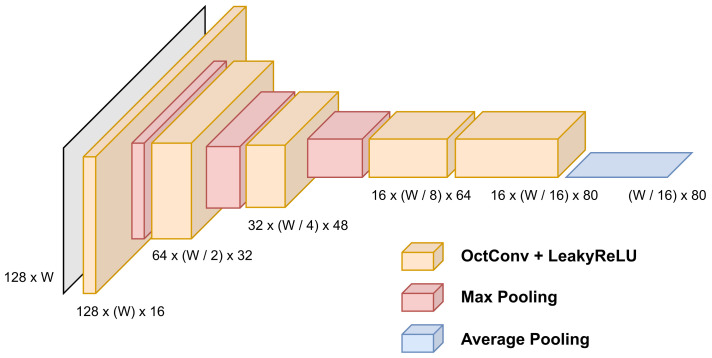
Feature maps produced by each layer of the Octave-CNN model.

The proposed Octave-CNN architecture, as shown in Figure 1, is aimed at the extraction of features from the input image in a feed-forward manner. It consists of five convolutional blocks, each one containing an OctConv layer with kernel size 3×3 pixels, stride equal to 1 and batch normalization [21]. The leaky rectified linear (LeakyReLU) [22] function is used for neuron activation, which provides a small gradient value when the unit is not active. A maximum pooling layer with kernel size equal to 2×2 is used after the first three blocks, to reduce the spatial dimensions of the features. Additionally, a dropout layer, with probability equal to 0.2 (experimentally defined) is included in the last three blocks, to assist for better generalization ability and robustness of the features [23]. The combination of batch normalization and dropout achieved best performance during our experimentation, which coincides with the findings of several state-of-the-art works [2,4,14]. Finally, the average of each column of the feature maps of the last layer is calculated, to acquire a feature vector with 80 features for each time step along the width of the image, as shown in Figure 3.

### 3.3. Recurrent BGRU Stage

**Figure 4:**
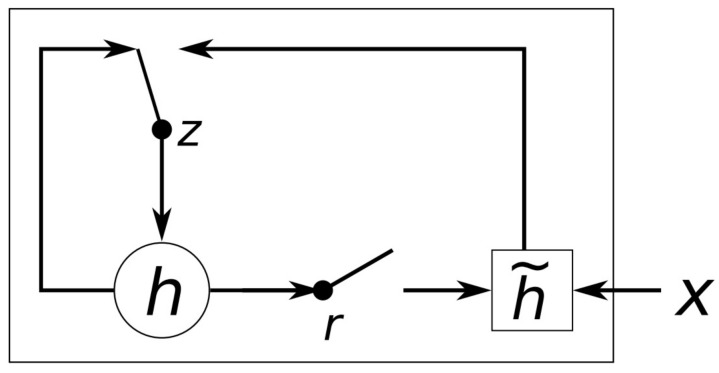
An illustration of the gated recurrent unit (GRU) [8].

The gated recurrent unit (GRU) [8] is a recurrent network architecture that comprises two gates, namely the reset gate *r* and the update gate *z*, as shown in Figure 4. For each recurrent unit *j* at time-step *t*, the current input $x^{t}$ and the activation $h^{t-1}$ of the previous time-step are used to compute both gates as follows:

(3) $$r_{j}=\sigma \left({\left[W_{r}x^{t}\right]}_{j}+{\left[U_{r}h^{t-1}\right]}_{j}\right)$$

(4) $$z_{j}=\sigma \left({\left[W_{z}x^{t}\right]}_{j}+{\left[U_{z}h^{t-1}\right]}_{j}\right)$$

where σ is the sigmoid activation function and $W_{r}$, $U_{r}$, $W_{z}$, $U_{z}$ denote trainable weights of the network. Subsequently, the reset gate *z* is used to compute a candidate activation, according to the following equation:

(5) $${\tilde{h}}_{j}^{t}=\tan h\left({\left[Wx^{t}\right]}_{j}+{\left[U\left(r⊙h^{t-1}\right)\right]}_{j}\right)$$

where **W**, **U** denote trainable weights and ⊙ denotes element-wise multiplication. The values of the reset gate determine the degree that the previous state affects the candidate output ${\tilde{h}}_{t}$, allowing the network to choose whether to retain or forget previously seen inputs. Finally, the update gate $z_{t}$ is used to compute the output $h_{t}$, as a linear interpolation between the previous output $h_{t-1}$ and the candidate output ${\tilde{h}}_{t}$:

(6) $$h_{j}^{t}=z_{j}h_{j}^{t-1}+\left(1-z_{j}\right){\tilde{h}}_{j}^{t}$$

In the proposed architecture, as shown in Figure 1, the recurrent stage contains three BGRUs, with 128 hidden units each, preceded by a dropout layer. Additionally, after each BGRU a fully connected layer is added to increase the complexity of the network. The first two fully connected layers comprise 256 neurons each, while the last one contains a number of neurons equal to the size of the character set of the minuscule writing, plus one for the blank symbol. The softmax activation function is also used to map neuron activations to classification probabilities.

## 4. Experimental Evaluation

### 4.1. Datasets

For the experimental evaluation of the proposed methodology we have considered three newly created collections of Greek historical handwritten documents, namely, χϕ53, χϕ79 and χϕ114, along with the “EPARCHOS” dataset [4,24]. Additionally, to enable comparison with the state of the art, we have included in our experimentation two public datasets: IAM [5] and RIMES [6]. Table 1 presents the details of each collection, i.e., the total number of pages, lines and words contained. A more detailed presentation regarding the newly created collections is also presented in the following sections.

#### 4.1.1. Stavronikita Monastery Collection No. 53 (χϕ53)

**Figure 5:**
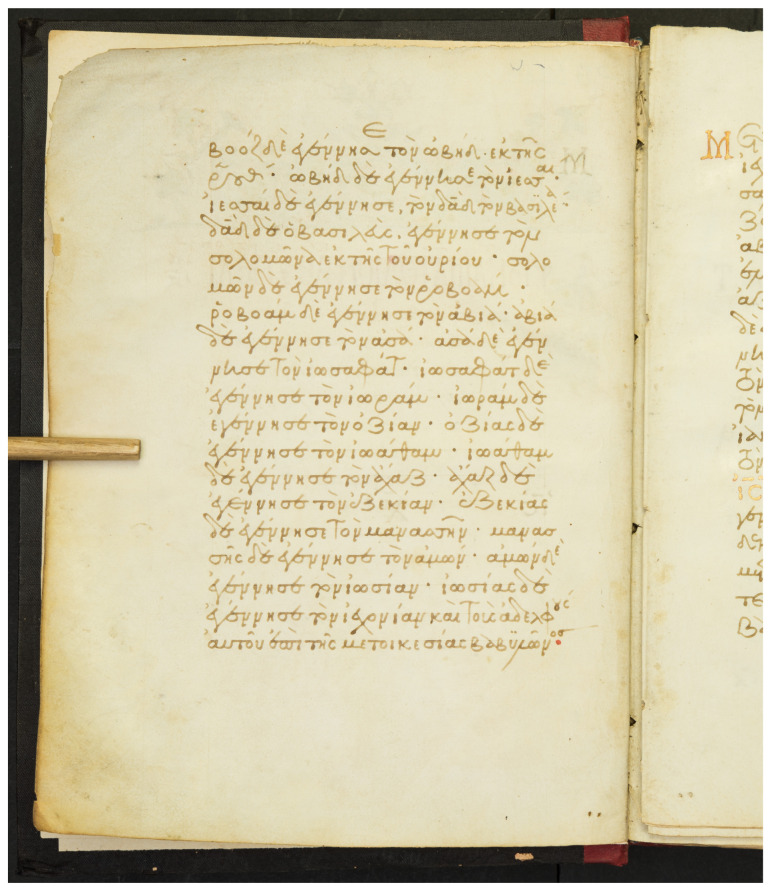
Example document image from the collection χϕ53.

The collection is one of the oldest Stavronikita Monastery on Mount Athos. It is a parchment four-gospel manuscript which has been written between 1301 and 1350. It comprises 54 pages with dimensions that are approximately 250 × 185 mm. The script is elegant minuscule and the use of majuscule letters is rare. Tachygraphical symbols and abbreviations are encountered in the manuscript as well. Furthermore, the manuscript is enriched with chrysography, elegant epititles and initials. The dataset of χϕ53 consists of 1038 text lines, containing 5592 words (2374 unique words) distributed over 54 scanned handwritten text pages. An example page is shown in Figure 5, and the collection is publicly available for research purposes [25].

#### 4.1.2. Stavronikita Monastery Collection No. 79 (χϕ79)

**Figure 6:**
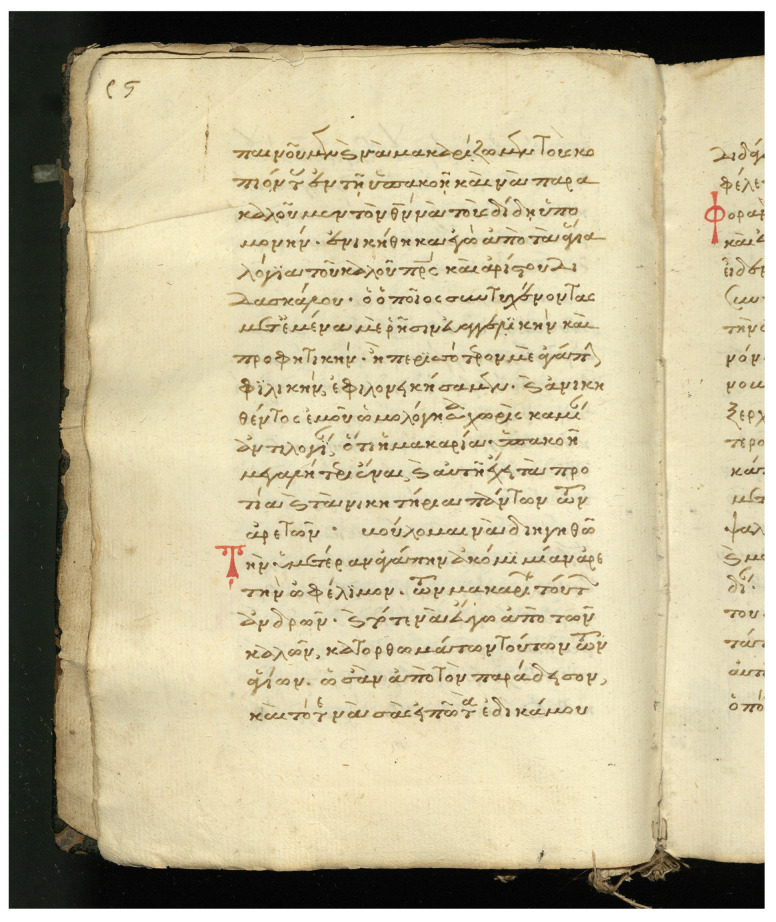
Example document image from the collection χϕ79.

The collection comprises manuscripts made of paper, written in the 16th century and its dimensions are 220 × 165 mm. The manuscript is embellished with epititles and red initials. Tachygraphical symbols and abbreviations are encountered in the manuscript as well. The dataset of χϕ79 consists of 803 text lines containing 4389 words (2069 unique words) distributed over 40 scanned handwritten text pages. An example page is shown in Figure 6, and the collection is publicly available for research purposes [26].

#### 4.1.3. Stavronikita Monastery Collection No. 114 (χϕ114)

**Figure 7:**
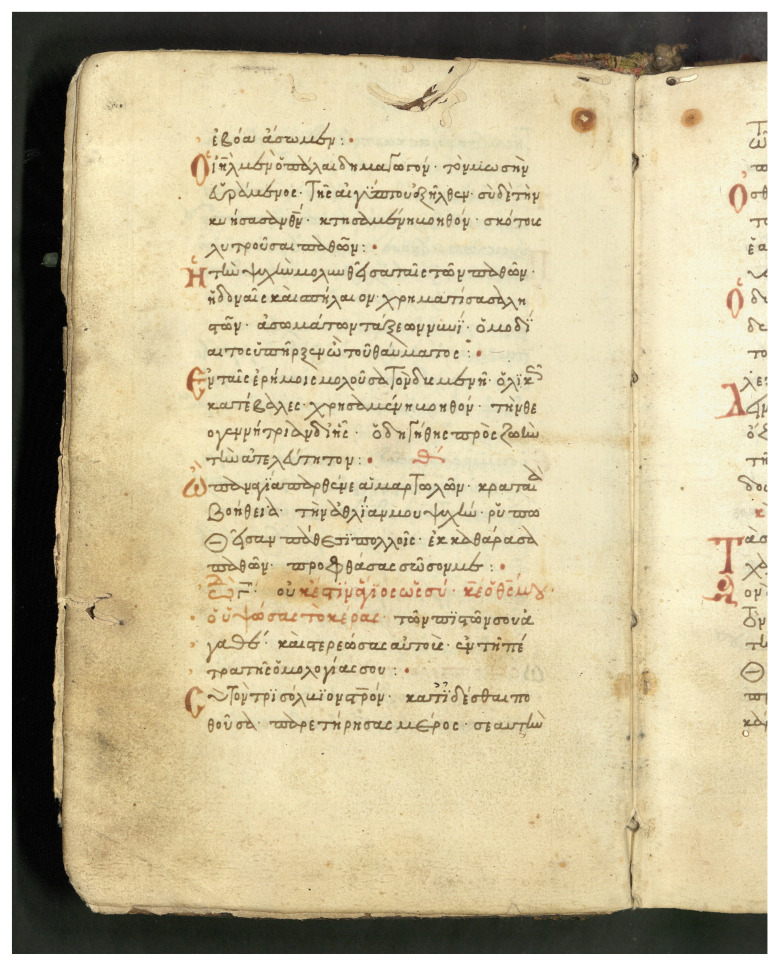
Example document image from the collection χϕ114.

The collection comprises manuscripts made of paper, written at the end of the 15th century and its dimensions are 218 × 150 mm. In various pages, we find red initials and epititles which enrich the manuscript’s decoration. The dataset of χϕ114 consists of 1051 text lines containing 5467 (2877 unique words) words that are distributed over 44 scanned handwritten text pages. An example page is shown in Figure 7, and the collection is publicly available for research purposes [27].

#### 4.1.4. Historical Greek ‘EPARCHOS’ Dataset

The dataset is a Greek historical handwritten codex by two writers, Antonius Eparchos and Camillos Zanettus. The Historical Greek “EPARCHOS” Dataset [24], which dates around 1500–1530, includes 120 scanned handwritten text pages and 9285 text lines containing 18,809 words (6787 unique words).

#### 4.1.5. IAM

The IAM (Institut für Informatik und Angewandte Mathematik) dataset [5] is an English written collection from 657 different writers with 1539 handwritten scanned text pages, consisted of 9000 text lines. Due to the grayscale color, the darkening throughout the words, and the transparent background of images, this collection offers a line recognition task independent of the writer, which means that each writer’s handwriting can only be discovered in a particular subset.

#### 4.1.6. RIMES

A challenging dataset due to numerous accented characters, but with a good quality background and precise writing is this of the database RIMES (Reconnaissance et Indexation de données Manuscrites et de fac similÉS) [6]. The database, written in French, consisted of over 12,000 text lines, 5600 handwritten mails, and sundry writers.

### 4.2. Particularities

In this section, we highlight the unique features arising from the minuscule writing, that are prominent in the Greek historical handwritten document collections. Each remark is accompanied with corresponding examples.

**Figure 8:**
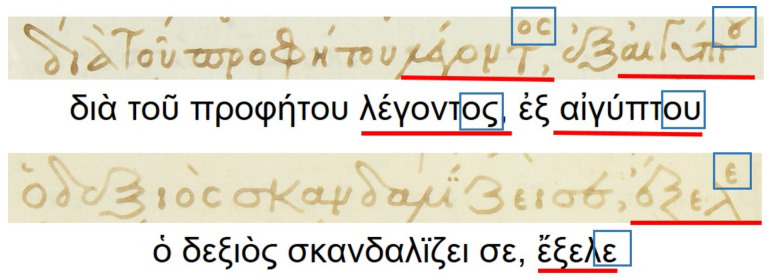
Floating characters appearing at word endings. The floating portion of the word is represented by a rectangle, while the rest of the word is underlined.

**Figure 9:**
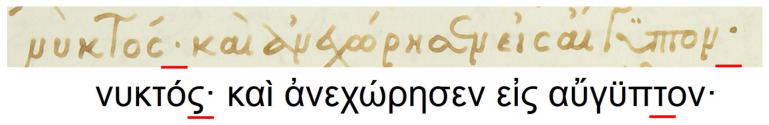
‘Minuscule’ writing example. Key locations in the text line that correspond to this particularity are underlined.

**Figure 10:**
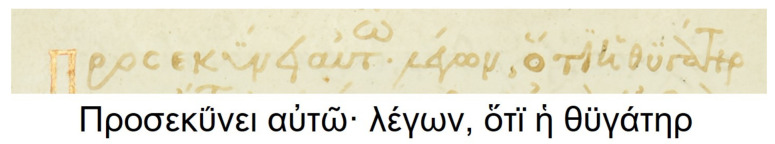
Polytonic orthography example.

- Floating characters: This is a common characteristic appearing in the word endings, where the last characters of the word are written in an abbreviated manner. Floating characters can appear both in uninflected or inflected words. Two examples of floating characters are shown in Figure 8.
- Minuscule writing: A notable distinction is the usage of a lowercase letter rather than an uppercase letter following a ’full stop’ character, as shown in the example of Figure 9. This is owing to the fact that there were no capital letters in use at the time, and this style of writing is known as ‘Minuscule’.
- Polytonic orthography: The polytonic system is common in all Byzantine manuscripts, as illustrated in the example document images shown in s Figure 5, Figure 6 and Figure 7. A particularity of this polytonic system are the characters greek ϊ and greek ϋ, which were used in this form to be distinguished from the diphthong letters, as shown in Figure 10. The problem with these characters concerns their transcription, which is not unique but it relies upon the context. In particular, either the character is transcribed as shown or transcribed as a character without the specific diacritic marks (diaeresis).

### 4.3. Experimental Setup

The four Greek historical handwritten document collections are proportionally partitioned into training, validation and testing sets with ratios 60%, 10% and 30%, respectively. The specific number of lines assigned to each partition are presented in Table 1. For the IAM and RIMES datasets we followed the corresponding training–testing partitioning provided by the creators, to enable comparison with the state of the art. For the experiments conducted, the Character Error Rate (CER) and the Word Error Rate (WER) are used as evaluation metrics to assess HTR performance.

For the training process, the RMSProp method [28] was used for gradient-descent optimization, with a learning rate of 0.001. The mini-batch size was set to 16 images while the input images were resized to a fixed height of 128 pixels. The training process was terminated if the progress, in terms of CER, was stalled for 20 consecutive epochs, when evaluating on the validation set. The values of these hyperparameters have been defined experimentally and they are kept the same for all the models tested. Decoding was performed using the ‘greedy’ method: during each time-step the class that corresponds to the logit with the maximum value is assigned. Subsequently, repeating characters are eliminated without the use of a language model. This enables comparison of the performance of the different models tested, minimizing the effect of a decoding scheme or a language model in performance.

All the experiments were conducted using the Tensorflow framework, running on a computer with Intel Core i7 4770 K processor, 32 GB memory and an NVidia Titan Xp GPU with 12 GB of available VRAM.

### 4.4. Results

As a preliminary step, we intend to evaluate the effect of preprocessing on the performance of the proposed HTR model in the four Greek historical handwritten datasets. The results presented in Table 2 demonstrate that the preprocessing is beneficial to at least three out of the four datasets with Greek historical handwritten documents. The similar performance achieved for the ‘EPARCHOS’ dataset in both cases is attributed to the fact that the writing in this collection is more clear compared to the other collections.

Next, comparison with state of the art is performed, taking into consideration two recently proposed methods by Puigcerver [2] and de Sousa Neto et al. [14].

In Table 3, the total number of parameters and the average training time per iteration are presented for dataset χϕ53. In Table 4, the results in terms of CER and WER are presented, where the numbers in bold represent the best result. It should be noted that the experimental results reported in Table 4, concerning the approaches of Puigcerver [2] have been produced using the PyLaia toolkit (available at https://github.com/jpuigcerver/PyLaia, accessed on 25 October 2021). Correspondingly, the results reported for the approach in [14] have been produced using an open-source implementation provided by the authors (available at https://github.com/arthurflor23/handwritten-text-recognition, accessed on 25 October 2021).

### 4.5. Discussion

As it is shown, considering the four collections of Greek historical handwritten documents, the proposed method outperformed the other two. On average, the difference between the proposed and the state-of-the-art methods equals to 3.5% and 6.4% for CER and WER, respectively. This can be attributed to improved feature extraction in the encoding part of the network due to the use of Octave convolutions. This becomes sound, in particular, considering the heavy use of diacritics in the Greek language of that period. It can be argued that it is beneficial to hierarchically extract and combine features in different scales via Octave convolutions. This leads to an increased ability of each layer capturing the subtle differences between the possible versions of the same character included in the character set.

**Figure 11:**
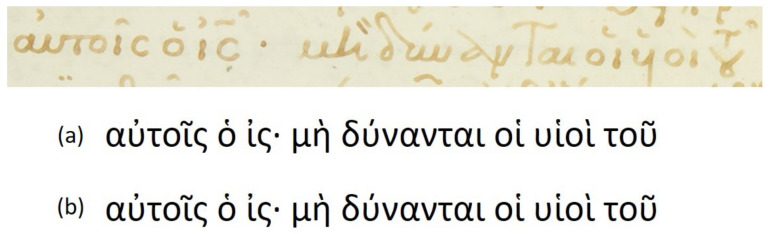
An example of a correctly predicted text line image along with the corresponding (**a**) groud-truth and (**b**) predicted texts.

**Figure 12:**
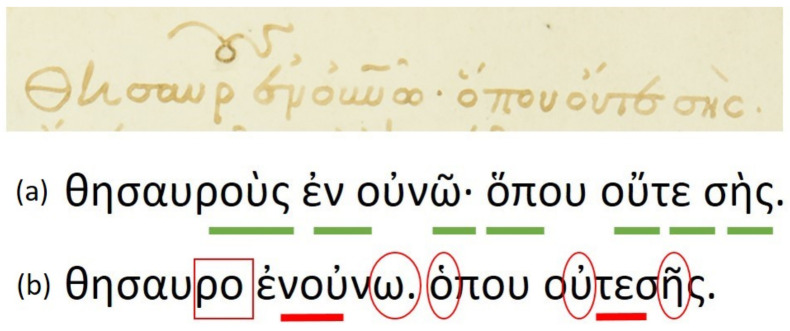
An example of a problematic text line image along with the corresponding (**a**) groud-truth and (**b**) predicted texts. The errors concern diacritics (circle), spacing (red line) and abbreviations (square).

Figure 11 and Figure 12 show examples of a correctly predicted and a problematic text line image, respectively, along with the corresponding ground-truth and predicted texts. In the latter, some of the most frequent prediction errors concern particularities of the language discussed in Section 4.2 and are pointed out.

## 5. Conclusions

In this paper, an OctCNN-BGRU architecture to address the problem of HTR in historical Greek manuscripts is detailed. Furthermore, a new collection of three historical Greek datasets for HTR is presented that is made publicly available for research purposes. The proposed model is shown to be better suited for the language of the specific era, outperforming the state-of-the-art approaches, on the Greek historical collections.

A limitation of this work lies in the fact that text line detection and segmentation in the document image is not addressed. It is also worth noting that the reported results have been achieved without the use of a language model. Future work involves the integration of the proposed architecture into an end-to-end pipeline for handwritten recognition that will process the raw manuscript image. Additionally, the construction of a language model along with a more elaborate decoding scheme will be an important aspect towards improving transcription performance, as indicated by the state-of-the-art research.

## Figures and Tables

**Table 1:** Characteristics of the datasets used for performance evaluation.

Dataset	Total Pages	Total Lines	Total Words	Training	Validation	Test
χϕ53	54	1038	5592	622	104	312
χϕ79	40	803	4389	481	80	242
χϕ114	44	1051	5467	603	100	302
Eparchos	120	2272	18809	1363	227	682
IAM	1539	8922	10,841	6161	900	1861
RIMES	1500	12,104	6358	10,193	1133	778

**Table 2:** Performance comparison of the proposed architecture operating with and without image preprocessing on the four Greek Historical collections. The best results for each dataset are indicated in bold.

Dataset	μDoc (Deslanting)	μDoc (no Deslanting)
χϕ53	**6.77/30.09**	7.19/31.22
χϕ79	**6.51/28.51**	6.73/28.21
χϕ114	**7.71/34.30**	8.32/36.44
Eparchos	4.53/20.03	**4.16/19.67**

**Table 3:** Total parameters and training time per item (ms), measured during experimentation with the χϕ53 collection.

HTR System	Parameters	Time per Iteration (ms)
Puigcerver	9,982,713	12.8
Flor	994,057	13.7
μDoc	2,246,137	8.0

**Table 4:** Performance comparison of the proposed architecture with two state of the art approaches on six datasets.The best results for each dataset are indicated in bold.

Dataset	de Sousa Neto et al. [14]	Puigcerver [2]	Proposed
χϕ53	7.85/34.63	10.45/30.20	**6.77/30.09**
χϕ79	7.75/33.13	10.33/28.55	**6.51/28.51**
χϕ114	8.03/36.72	10.19/34.58	**7.71/34.30**
Eparchos	4.95/21.91	5.18/22.21	**4.53/20.03**
IAM	7.32/24.19	**6.49/20.91**	7.30/23.72
RIMES	6.65/28.31	**3.76/12.60**	6.52/29.49

## Data Availability

The EPARCHOS dataset is openly available in Zenodo at https://doi.org/10.5281/zenodo.4095301 (accessed on 25 October 2021), reference number [25]. The Stavronikita Monastery Greek Handwritten Document Collection No. 53 (χϕ53) is openly available in Zenodo at https://doi.org/10.5281/zenodo.5595669 (accessed on 25 October 2021), reference number [25]. The Stavronikita Monastery Greek Handwritten Document Collection No. 79 (χϕ79) is openly available in Zenodo at https://doi.org/10.5281/zenodo.5578136 (accessed on 25 October 2021), reference number [26]. The Stavronikita Monastery Greek Handwritten Document Collection No. 114 (χϕ114) is openly available in Zenodo at https://doi.org/10.5281/zenodo.5578251 (accessed on 25 October 2021), reference number [27].

## References

[1] De Sousa Neto A.F., Bezerra B.L.D., Toselli A.H., Lima E.B. HTR-Flor++: A Handwritten Text Recognition System Based on a Pipeline of Optical and Language Models. Proceedings of the DocEng ’20: ACM Symposium on Document Engineering 2020.

[2] Puigcerver J. Are Multidimensional Recurrent Layers Really Necessary for Handwritten Text Recognition?. Proceedings of the 14th IAPR International Conference on Document Analysis and Recognition.

[3] Chen Y., Fan H., Xu B., Yan Z., Kalantidis Y., Rohrbach M., Yan S., Feng J. Drop an Octave: Reducing Spatial Redundancy in Convolutional Neural Networks with Octave Convolution. Proceedings of the 2019 IEEE/CVF International Conference on Computer Vision.

[4] Markou K., Tsochatzidis L.T., Zagoris K., Papazoglou A., Karagiannis X., Symeonidis S., Pratikakis I. A Convolutional Recurrent Neural Network for the Handwritten Text Recognition of Historical Greek Manuscripts. Proceedings of the Pattern Recognition, ICPR International Workshops and Challenges.

[5] Marti U., Bunke H. (2002). The IAM-database: An English sentence database for offline handwriting recognition. Int. J. Doc. Anal. Recognit..

[6] Grosicki E., Carré M., Brodin J., Geoffrois E. Results of the RIMES Evaluation Campaign for Handwritten Mail Processing. Proceedings of the 10th International Conference on Document Analysis and Recognition.

[7] Hochreiter S., Schmidhuber J. (1997). Long short-term memory. Neural Comput..

[8] Cho K., Van Merriënboer B., Gulcehre C., Bahdanau D., Bougares F., Schwenk H., Bengio Y. (2014). Learning phrase representations using RNN encoder-decoder for statistical machine translation. arXiv.

[9] Voigtlaender P., Doetsch P., Ney H. Handwriting Recognition with Large Multidimensional Long Short-Term Memory Recurrent Neural Networks. Proceedings of the 15th International Conference on Frontiers in Handwriting Recognition.

[10] Graves A., Schmidhuber J. (2008). Offline handwriting recognition with multidimensional recurrent neural networks. Adv. Neural Inf. Process. Syst..

[11] Pham V., Bluche T., Kermorvant C., Louradour J. Dropout Improves Recurrent Neural Networks for Handwriting Recognition. Proceedings of the 14th International Conference on Frontiers in Handwriting Recognition.

[12] Bluche T., Messina R.O. Gated Convolutional Recurrent Neural Networks for Multilingual Handwriting Recognition. Proceedings of the 14th IAPR International Conference on Document Analysis and Recognitionf.

[13] Dauphin Y.N., Fan A., Auli M., Grangier D. Language modeling with gated convolutional networks. Proceedings of the International Conference on Machine Learning.

[14] De Sousa Neto A.F., Bezerra B.L.D., Toselli A.H., Lima E.B. HTR-Flor: A Deep Learning System for Offline Handwritten Text Recognition. Proceedings of the 33rd SIBGRAPI Conference on Graphics, Patterns and Images.

[15] Retsinas G., Sfikas G., Nikou C. Iterative Weighted Transductive Learning for Handwriting Recognition. Proceedings of the 16th International Conference on Document Analysis and Recognition.

[16] Kang L., Riba P., Rusiñol M., Fornés A., Villegas M. (2020). Pay Attention to What You Read: Non-recurrent Handwritten Text-Line Recognition. arXiv.

[17] Wick C., Zöllner J., Grüning T. Transformer for Handwritten Text Recognition Using Bidirectional Post-decoding. Proceedings of the 16th International Conference on Document Analysis and Recognition.

[18] Wick C., Zöllner J., Grüning T. (2021). Rescoring Sequence-to-Sequence Models for Text Line Recognition with CTC-Prefixes. arXiv.

[19] Chen K., Chen C., Chang C. (2012). Efficient illumination compensation techniques for text images. Digit. Signal Process..

[20] Vinciarelli A., Luettin J. (2001). A new normalization technique for cursive handwritten words. Pattern Recognit. Lett..

[21] Ioffe S., Szegedy C. Batch normalization: Accelerating deep network training by reducing internal covariate shift. Proceedings of the International Conference on Machine Learning.

[22] Maas A.L., Hannun A.Y., Ng A.Y. (2013). Rectifier Nonlinearities Improve Neural Network Acoustic Models. https://citeseerx.ist.psu.edu/viewdoc/download?doi=10.1.1.693.1422&rep=rep1&type=pdf.

[23] Srivastava N., Hinton G., Krizhevsky A., Sutskever I., Salakhutdinov R. (2014). Dropout: A simple way to prevent neural networks from overfitting. J. Mach. Learn. Res..

[24] Papazoglou A., Pratikakis I., Markou K., Tsochatzidis L. (2020). EPARCHOS - Historical Greek Handwritten Document Dataset. (1.0) [Data Set]. Zenodo. https://zenodo.org/record/4095301#.YaneeroxVPY.

[25] Pratikakis I., Papazoglou A., Symeonidis S., Tsochatzidis L. (2021). Stavronikita Monastery Greek Handwritten Document Collection No. 53. (1.0) [Data Set]. Zenodo. https://zenodo.org/record/5595669#.YaneoLoxVPY.

[26] Pratikakis I., Papazoglou A., Symeonidis S., Tsochatzidis L. (2021). Stavronikita Monastery Greek Handwritten Document Collection No. 79. (1.0) [Data Set]. Zenodo. https://zenodo.org/record/5578136#.YaneuboxVPY.

[27] Pratikakis I., Papazoglou A., Symeonidis S., Tsochatzidis L. (2021). Stavronikita Monastery Greek Handwritten Document Collection No. 114. (1.0) [Data Set]. Zenodo. https://zenodo.org/record/5578251#.YaneyboxVPY.

[28] Tieleman T., Hinton G. (2012). Lecture 6.5-rmsprop: Divide the gradient by a running average of its recent magnitude. Coursera Neural Netw. Mach. Learn..

